# River–sea thermal differential experienced by salmon post‐smolts is not a proximal driver of marine survival

**DOI:** 10.1111/jfb.70134

**Published:** 2025-07-08

**Authors:** Emma Tyldesley, Neil Banas, Richard Kennedy, Graeme Diack, Colin Bull

**Affiliations:** ^1^ Department of Mathematics and Statistics University of Strathclyde Glasgow UK; ^2^ Atlantic Salmon Trust Perth UK; ^3^ Agri‐Food and Biosciences Institute Aquatics Group River Bush Salmon Station Bushmills UK; ^4^ Missing Salmon Alliance Kilgraston UK; ^5^ Aquaculture, Faculty of Natural Science, Institute of Aquaculture University of Stirling Stirling UK

**Keywords:** Atlantic salmon, indicators, marine survival, migration, phenology, *Salmo salar*

## Abstract

Wild Atlantic salmon (*Salmo salar*) populations from many North Atlantic rivers have declined over recent decades. As these declines are thought to be driven largely by changes in the marine phase of the life cycle, there is a drive towards investigating causes and developing indicators for marine survival. Warming of rivers and seas is altering the smolt thermal environments and migration phenology. This may be causing increasing mismatch between in‐river cues used by salmon to optimise the timing of their downstream migration and the suitability of thermal and trophic conditions encountered on sea entry. In this study, recently mobilised data on smolt migration timing, adult marine return rates and freshwater and marine temperatures are used to assess the potential of river–sea thermal differential as a driver of marine survival for a set of southern European Atlantic salmon populations. Shifts in smolt migration phenology appear to have buffered these populations against increasing freshwater temperatures, but post‐smolts are migrating into a warming coastal environment. There was no evidence for significant trends in river–sea entry thermal differential, and a significant correlation with marine return rates was found for only one of the seven study populations. For these populations, this suggests that thermal differential at smolting is not a consistent driver or predictor of marine return rates and would not form the basis of a generally applicable indicator of marine survival.

## 1 INTRODUCTION

Wild Atlantic salmon (*Salmo salar*) populations from many North Atlantic rivers have declined over the last few decades (ICES, 2023; Olmos et al., 2019). Although management action is often focused on the freshwater stage of the salmon life cycle, the continuing population declines are thought to be driven by reduced marine survival (Chaput, 2012; Olmos et al., 2019; Thorstad et al., 2012). This has led to calls for the evaluation of potential ‘likely suspects’ and development of indicators of salmon marine survival (Bull et al., 2022; ICES, 2020). Survival has been linked with marine variables, including large‐scale climate indices (Beaugrand et al., 2003; Beaugrand & Reid, 2013; Jensen et al., 2011;Mills et al., 2013; Olmos et al., 2020), temperature (Friedland et al., 1998, 2000, 2003; Olmos et al., 2020), primary productivity (Mills et al., 2013; Olmos et al., 2020) and zooplankton energy (Beaugrand & Reid, 2013; Tyldesley et al., 2024), but the challenge remains to find consistent, ecologically founded and usable relationships (Wainwright, 2021). This study assesses the potential of river–sea temperature differential experienced by post‐smolts entering the marine environment as both a driver and an operational indicator of marine survival.

Smolting refers to the physical and behavioural adaptations undertaken by salmon parr to prepare for downstream migration, seawater entry and marine residence (McCormick et al., 1998). Fish become silvery and streamlined, lose their positive rheotactic behaviour and increase their saltwater tolerance, olfactory sensitivity, metabolic rate, growth capacity and schooling tendency (McCormick et al., 1998). This change is primed by individual body size, changing photoperiod and accumulated thermal environment (McCormick et al., 1998; Thorstad et al., 2012). Downstream migration is then triggered by more proximal factors – primarily temperature, but also flow, lunar phase and social cues (Antonsson & Gudjonsson, 2002; Hvidsten et al., 1995; Kennedy & Crozier, 2010; McCormick et al., 1998). The timing of smolt migration (‘the smolt run’) therefore varies regionally and interannually (Otero et al., 2014) and is considered to be a population‐specific adaptation using in‐river cues to optimise the timing of sea entry for favourable thermal and trophic marine conditions (Antonsson & Gudjonsson, 2002; Hvidsten et al., 1998; McCormick et al., 1998, 2009; Satterthwaite et al., 2014).

Smolting and sea entry are critical periods in the salmon life cycle, with high and variable mortality as the fish adapt to salt water and pass through the high predation environment of the river mouth or estuary (Thorstad et al., 2012). Smolts attempt to avoid predation by schooling, migrating together within a 1–2‐week pulse, travelling at night and spending only a brief period in the transition environment without a significant acclimation period (Lilly et al., 2022; McCormick et al., 1998; Moore et al., 1995; Thorstad et al., 2012). Any factor that causes a shift in smolt migration timing away from the optimal window, delaying fish at the river mouth or reducing their ability to feed and avoid predators, has the potential to increase mortality (McCormick et al., 2009).

Climate change is causing warming of rivers and seas (IPCC, 2021; Loerke et al., 2023). Warmer springs are associated with earlier smolt migration (e.g., Antonsson & Gudjonsson, 2002; Hvidsten et al., 1995; Jutila et al., 2009). There is evidence that climate change is advancing the start of the smolt run by several days per decade (de Eyto et al., 2022; Jonsson & Jonsson, 2009; Kennedy & Crozier, 2010; Kovach et al., 2013; Otero et al., 2014; Russell et al., 2012; Simmons et al., 2021). Sea surface temperature (SST) fluctuations in the early marine phase have been linked to changes in post‐smolt behaviour and survival (Friedland et al., 1998, 2003; Hvidsten et al., 2009; Jutila et al., 2005; Olmos et al., 2020). At the same time, ocean phenology and ecosystems are changing, with advances in the timing of the spring plankton bloom and shifts in species composition, abundance and size structure (Capuzzo et al., 2018; Edwards et al., 2001, 2021; Henson et al., 2009; Schmidt et al., 2020; Tyldesley et al., 2024). The combination of these freshwater and marine changes may result in increasing decoupling of in‐river cues for migration from suitable thermal and trophic ocean conditions encountered by post‐smolts (Jonsson & Jonsson, 2009; McCormick et al., 2009; Russell et al., 2012; Thorstad et al., 2012).

As climate change is expected to increase temperatures more rapidly over land than sea (IPCC, 2021), freshwater temperatures experienced by smolts are expected to warm faster than the coastal waters into which they migrate. On the River Bush, Northern Ireland, the thermal difference between fresh and salt water during the smolt run was found to increase significantly over 1978–2008. This thermal difference was found to be negatively correlated with adult marine return rates (Kennedy & Crozier, 2010). Thermal difference may be a direct stressor, as unsuitable water temperatures can affect salmon directly by influencing their metabolism, performance and behaviour (Brett, 1971). Salmon have a thermal tolerance zone and can experience stress outside this range depending on the exposure time, rate of change and temperature to which the fish is acclimatised (Jonsson & Jonsson, 2009). As fish move from fresh to salt water, a large, sudden temperature change has been shown to reduce swimming performance and the ability to adjust to salt water (Brett, 1971; Staurnes et al., 2001). The thermal difference may also be a proxy for suitability of marine trophic conditions, that is, degree of match–mismatch between smolt migration and the phenology of their prey (Cushing, 1975), which has been shown to affect marine survival in some Pacific salmonid populations (Satterthwaite et al., 2014; Wilson et al., 2021).

To date, no broad‐scale investigation has examined the relationship between river–sea thermal difference during smolt migration and marine survival across Atlantic salmon populations. Previous work on general trends in river–sea thermal differential has been hampered by low spatial and temporal resolution of marine temperature data (e.g., ICES, 2010) In this study, we use newly mobilised data on smolt run timing, concurrent freshwater temperature and adult marine return rates for a set of monitored southern European salmon populations, combined with SST derived from an ocean model hindcast with high coastal resolution, to test whether sea entry thermal differential can predict marine return rates and therefore be a useful indicator of marine survival.

## 2 METHODS

### 2.1 Studied populations

**FIGURE 1:**
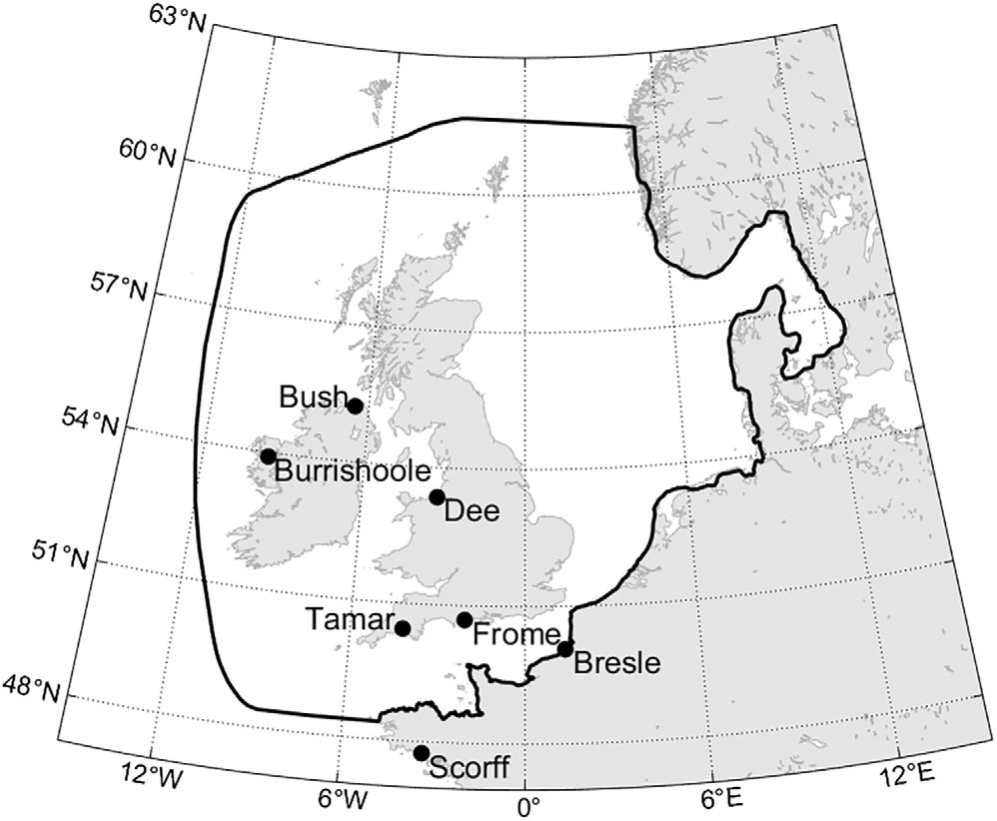
Locations of study salmon populations. Solid black line indicates the boundary of the Scottish Shelf Model domain.

This study used time series of annual smolt migration timing and adult return rate (i.e., the fraction of emigrating smolts that return to the river after one or more years) for a set of highly monitored salmon populations from southern Europe. These populations were from the River Bush in Northern Ireland, River Burrishoole in Ireland, River Tamar and River Frome in England, River Dee in Wales and River Bresle and River Scorff in France (Figure 1). The data are derived from fish tagging and trapping as part of co‐ordinated National Salmon Population Monitoring Programmes (ICES, 2024).

### 2.2 Adult marine return rates

The method for estimating numbers of returning adult salmon varied by river but involved either a full or a corrected partial count using a fish trap or counter in all cases (Tyldesley et al., 2024). Marine return rates are the percentage of migrating smolts in a population that return as adults after either one sea winter (1SW) or multiple sea winters (MSW, which aggregates fish spending 2 or more years at sea as per international stock assessment methods). This study used the annual 1SW return rates (ICES, 2023) (time series in Supplementary Information Figure 2) because 1SW fish make up the majority of returners in these study populations; the number of MSW fish is small and therefore the MSW return rates can be highly variable and uncertain.

**FIGURE 2:**
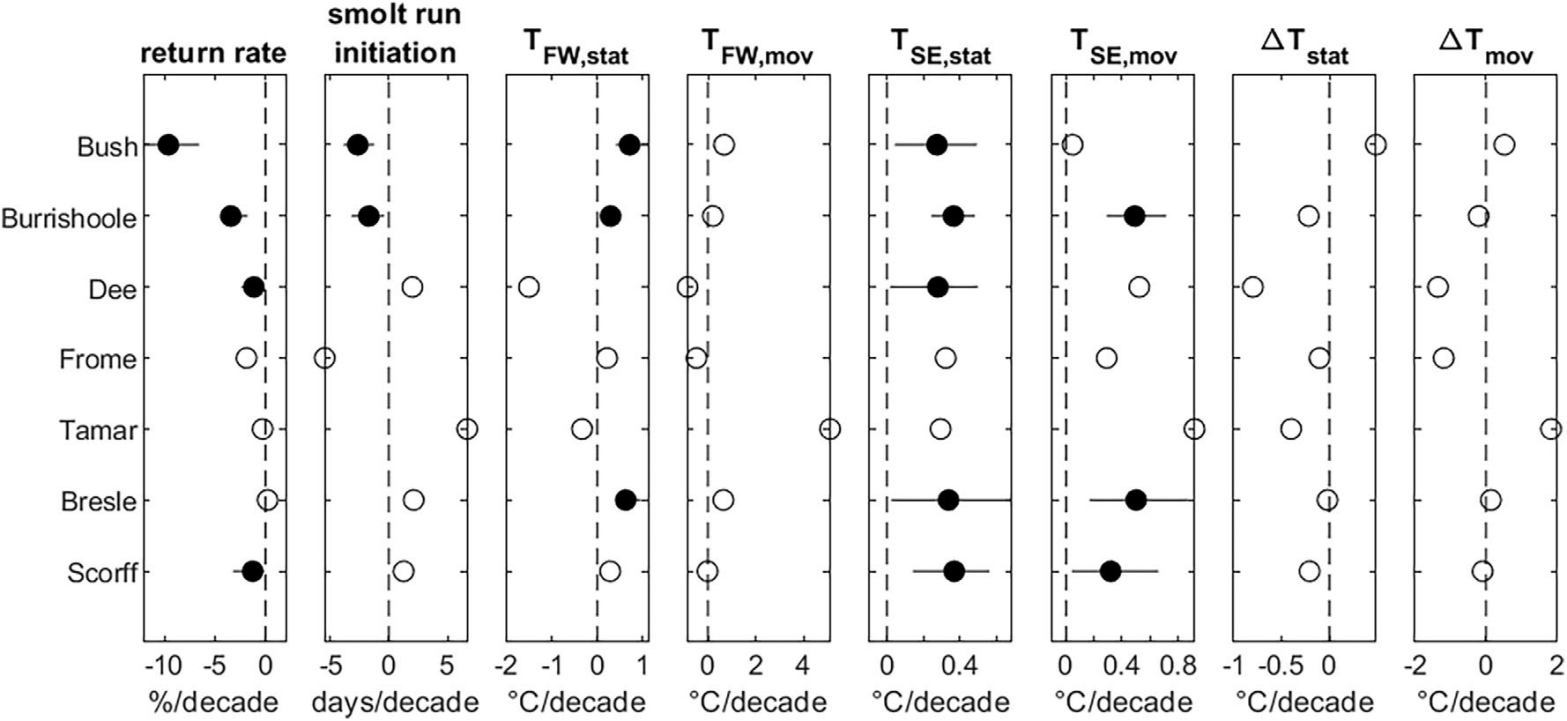
Trends in one sea winter adult return rates, smolt run initiation and smolt thermal environment indicators – static and moving freshwater temperature (*T*_FW,stat_ and *T*_FW,mov_), static and moving sea entry temperature (*T*_SE,stat_ and *T*_SE,mov_) and the difference between the two (∆*T*_stat_ and ∆*T*_mov_). Solid circles with 95% confidence intervals indicate trends with *p*‐value < 0.05. Open circles indicated *p*‐value≥0.05.

### 2.3 Onset of the smolt migration

The method of counting migrating smolts for each river was either a full count involving diverting migrating smolts from the main river, or a partial count, which was then raised to a full count using mark‐and‐recapture to estimate catch efficiency (ORE DiaPFC, 2024; Tyldesley et al., 2024). The length of the dataset varied by population, with the longest from the Burrishoole for 1970–2021. Following previous work, the onset of smolt migration each year was defined as the yearday on which 25% of the total smolts had been counted (Kennedy & Crozier, 2010; Otero et al., 2014) (time series in Figure S1). From the 90 percentile range of the onset dates we define a ‘typical onset month’ over the whole time series: April for the Scorff, Bresle, Tamar and Frome, and mid‐April to mid‐May for the Bush, Burrishoole and Dee (Tyldesley et al., 2024).

### 2.4 Metrics of smolt thermal environment

#### 2.4.1 River water temperature

Daily river water temperature measurements collected at the smolt trapping site for each river were obtained as summarised in Table 1. In all cases, these data were collected above the head of tide and therefore represent freshwater temperatures concurrent with the smolt migration. The time span varies by river.

**TABLE 1:** Details of daily river water temperature data.

River	Dates	Sampling details	Organisations	References
Bresle	1984–2021	Sampling location at returning adult sampling station ~3 km upstream of river mouth.	Environmental Research Observatory (ORE) on diadromous fish in coastal rivers (DiaPFC), France; Institute for Agriculture, Food and Environment, France (INRAE)	Flesselle et al. (2024)
Burrishoole	1960–2021	Sampling at same location as smolt trap: Mill Race channel between brackish Loch Furnace and freshwater Lough Feeagh. 1960–2004: Negretti paper chart recorder, calibrated against a mercury thermometer. 2004–2009: TidbiT temperature data logger. 2009–present: temperature sensor on an Orpheus Mini Water Level Recorder.	Marine Institute, Ireland	de Eyto et al. (2022) Dillane et al. (2024)
Bush	1990–2022	Sampling at same location as smolt and adult trap: River Bush Salmon Station, ~1 km upstream of river mouth.	The Agri‐Food and BioSciences Institute, Northern Ireland	Kennedy and Crozier (2010)
Welsh Dee	2002–2021	Sampling at same location as smolt and adult trap: Manley Hall weir ~50 km upstream.	Natural Resources Wales	
Frome	1992–2022	Sampling at same location as smolt and adult trap: on main river at East Stoke 8 km upstream of river mouth. Squirrel logger 1991–2009. Tinytag 2009–present.	Game and Wildlife Trust; John Davy‐Bowker River Laboratory	
Scorff	1995–2021	Sampling at same location as smolt and adult fish trap: Moulin des Princes Station at the limit of tidal influence ~15 km from river mouth. Tidbit temperature data logger.	ORE DiaPFC; INRAE	Jeannot (2024)
Tamar	2015–2021	Sampling at same location at smolt and adult fish trap: Gunnislake Weir at head of tide.	Environment Agency, England	

Two time series of the annual smolt freshwater thermal environment were calculated: a stationary measure *T*_FW,stat_ calculated as the mean temperature over the typical onset month for each river as defined above, and a non‐stationary measure *T*_FW,mov_ calculated as the mean temperature over the 5 days preceding the onset of the smolt migration in each year. The latter represents the thermal environment associated with the initiation of the smolt migration.

#### 2.4.2 Sea entry temperature

Sea entry temperatures were taken from the Scottish Shelf Waters Reanalysis Service 3.02 (SSW‐RS), a hydrodynamic model hindcast for the Scottish continental shelf and surrounding waters run for 1993–2019 (Figure 1) (Barton et al., 2022). This model is particularly suitable for this application because of its high coastal resolution assimilation of satellite SST to improve fit with historical data and annually varying riverine inputs dataset (Barton et al., 2024; O'Hara Murray, 2017). The model is run on the Scottish Shelf Model domain, which has an irregular grid with horizontal resolution from 0.5 km near the coast to 20 km in the open ocean and a hybrid depth layer scheme with fixed surface (3, 9, 16, 23 and 30 m below the surface) and deep (6 and 12 m above the seabed) bands in water shallower than 120 m and terrain following bands elsewhere. The riverine volume flux and temperature forcing data were obtained by merging climatology model data for Scottish and Northern Irish rivers (Centre for Ecology Grid2Grid, Bell et al., 2018) and daily model data for the whole domain (European‐Hydrological Predictions for the Environment, E‐HYPE, Arheimer et al., 2020).

For each river, a time series of annual sea entry temperature, defined as the mean within the model surface layer within 5 km of the river mouth, was calculated for the smolt run day (*T*_SE,mov_) and as an average over the typical onset month (*T*_SE,stat_). As it is not obvious which averaging distance would best represent ‘sea entry’ conditions, sensitivity analysis was carried out using values from 0.5 to 5 km. As this made at most 0.5°C difference to the sea entry temperature, 0.7°C difference to the thermal differential and no difference to the significance of trends or correlations analysis (see below), the conclusions of this study were unchanged. For the Scorff, which lies outside the SSW‐RS domain, SST data were extracted for the closest node, approximately 100 km northwest of the river (2–10 days of active swimming; Borland et al., 2024; Lilly et al., 2023). SST at this distance may not equal the SST at sea entry but is likely to exhibit similar interannual variability. Therefore, the study results are unlikely to be substantially affected.

#### 2.4.3 River–sea temperature differential

Time series of the annual sea entry temperature differential were then calculated during the smolt run, ∆*T*_mov_ = *T*_FW,mov_‐*T*_SE,mov_, and during the typical onset month, ∆*T*_stat_ = *T*_FW,stat_‐*T*_SE,stat_.

#### 2.4.4 Statistical analysis

The presence of trends in the time series of annual return rates, smolt run initiation timing and thermal indicators was determined using the non‐parametric Theil‐Sen estimator using the *sen_slope* function in Matlab (Afruzi, 2024). The Theil‐Sen estimator is robust to outliers, non‐normal data and heteroscedasticity (Ohlson & Kim, 2015; Sen, 1968; Theil, 1992).

Correlations of marine return rates with smolt run timing and the thermal indices were calculated using Pearson's correlation coefficient *r* with the modified Chelton method test for significance, which accounts for autocorrelation (Pyper & Peterman, 1998). Correlations in interannual fluctuations were measured by calculating Pearson's correlation *r*_diff_ of the *difference* in return rates between consecutive years, which acts to detrend the time series.

Previous work has sometimes found that relationships between salmon return rates and thermal indicators can vary with the time window analysed (e.g., Harvey et al., 2022; Wainwright, 2021). It can therefore be informative to carry out the statistical analysis using both full time series and restricting to an overlapping period for all populations. This is not possible here due to the variable sampling, which means there are very few fully overlapping periods for all populations (see Data S1). This study therefore treats the populations as independent and tests whether thermal metrics are useful across the full period of available data.

All analysis was carried out using the MATLAB numerical computing environment (The Mathworks Inc, 2021).

## 3 RESULTS

### 3.1 Trends in return rates, smolt phenology and thermal indicators

Marine return rates (hereafter ‘return rates’) for 1SW salmon during the study period exhibited significant declining trends for four populations during the study period: Bush (−9.6%/decade), Burrishoole (−3.4%/decade), Dee (−1.2%/decade) and Scorff (−1.3%/decade) (Figure 2).

There was a significant trend to earlier smolt run initiation for two populations: Bush (−2.7 days/decade; 12 days over 1978–2020) and Burrishoole (−1.7 days/decade; 9 days from 1970 to 2022). There were no significant trends for the Bresle, Dee, Frome, Tamar or Scorff populations over the study period.

Freshwater temperature in the typical onset month, *T*_FW,stat_, significantly increased for the Bush (0.5°C/decade), Burrishoole (0.3°C/decade) and Bresle (0.6°C/decade) populations. However, there were no significant trends in the freshwater temperature in the week prior to the initiation of the smolt migration in each year, *T*_FW,mov_, for any populations.

Sea entry temperature in the typical onset month, *T*_SE,stat_, significantly increased for most populations: Bush, Burrishoole, Dee, Bresle and Scorff (0.3–0.4°C/decade for each population). There was also a significant increase in sea entry temperature on the smolt migration initiation day for the Bush (0.2°C/decade), Burrishoole (0.5°C/decade), Bresle (0.6°C/decade) and Scorff (0.3°C/decade) populations.

There were no significant trends in river–sea thermal differential during the typical onset month, ∆*T*_stat_, or on the smolt run initiation day, ∆*T*_mov_, for any population.

### 3.2 Correlation between marine return rates and thermal indicators

**FIGURE 3:**
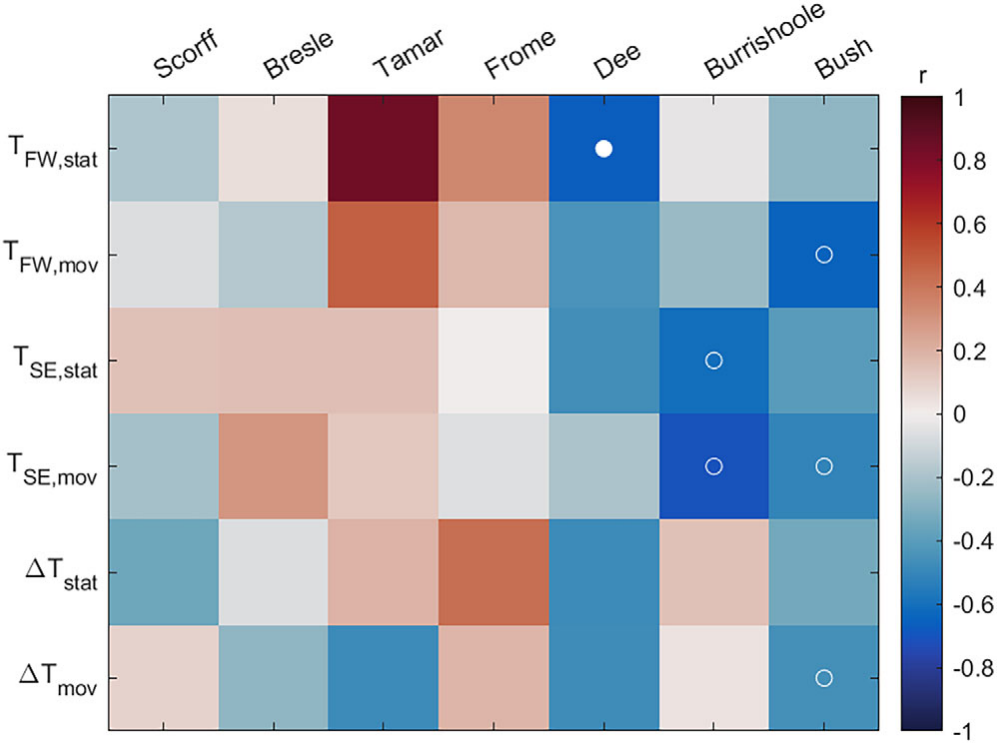
Correlation coefficient *r* of adult one sea winter (1SW) marine return rates with smolt thermal environment indicators by salmon population. Open circles indicate autocorrelation‐adjusted *p*_MC_ < 0.05. Filled circles indicate detrended *p*_diff_ < 0.05.

The analysis found few significant correlations between the smolt thermal environment indicators and the adult return rates (Figure 3). Return rates were significantly correlated with *T*_FW,stat_ for the Dee population for both raw and detrended time series and with *T*_FW,mov_ for the Bush population but not when detrended. Return rates were significantly correlated with *T*_SE,stat_ on the Burrishoole and with *T*_SE,mov_ on the Burrishoole and Bush but not when detrended.

Return rates for the Bush population were significantly correlated with ∆*T*_mov_ but not when detrended. There were no other significant correlations between return rates and ∆*T*_stat_ or ∆*T*_mov_.

## 4 DISCUSSION

This study found that smolt migration phenology has changed for some but not all the studied populations. There was a significant trend towards earlier migration for the Bush and Burrishoole. The other populations had a mix of non‐significant positive and negative trends. The variation in magnitude and direction of change may be related to variation between populations in the time period covered by the smolt run data (Harvey et al., 2022), as well as regionally varying responses to climate warming, as identified by other studies (Olmos et al., 2020; Vollset et al., 2022). Previous work has generally identified a shift towards earlier migration (e.g., Russell et al., 2012). Otero et al. (2014) calculated a trend in smolt run initiation of −2.5 days/decade for North Atlantic populations as a whole. Previous work on the Burrishoole population found a significant shift in smolt run onset (defined as yearday by which 5% of total smolt run had been counted rather than 25% as used here) of −1.8 days/decade over 1970–2020 (de Eyto et al., 2022). This is similar to the value of −1.6 days/decade found for the Burrishoole in this study using data over 1970–2022. Previous work on the Bush population found a significant shift in migration onset of −3.6 days/decade over 1978–2008 (Kennedy & Crozier, 2010). Extending the time series to 2022, this study found a trend of −2.7 days/decade, suggesting that the trend has slowed down in the recent decades. Using smolt counts over the whole migration period, Simmons et al. (2021) found a trend towards earlier migration over 2006–2022 for the Frome. Here, using only the day of the 25th percentile of smolt count each year as an indication of migration initiation, we found a non‐significant trend towards earlier migration (the slope between year and timing was negative but the confidence interval included zero).

At the same time, freshwater temperature during the average smolt run month has increased for most populations, but freshwater temperature at the time of the smolt run initiation has remained constant. This suggests that shifting phenology has acted to buffer these populations against experiencing a warming smolt environment. In contrast, sea entry temperature has increased for most populations using both the static and moving metric, that is, post‐smolts are migrating into a warming coastal zone.

Our study found no evidence for a trend in river–sea entry thermal differential for any populations contrary to the expected differential warming under climate change. The only significant correlation of thermal differential with marine return rates was found for the River Bush, confirming a continuation of the relationship identified up to 2008 by Kennedy and Crozier (2010). It is unclear why the relationship existed only for this population, but it may be related to the particular morphology of that river, which lacks an estuary and may leave its migrating salmon open to a different set of mortality pressures (Flávio et al., 2020) – the river discharges directly into the ocean across a steep, narrow stony beach. This may result in a sharp, steep temperature contrast, which has been shown to exacerbate thermal and osmotic stress (Staurnes et al., 2001). In addition, there will be intra‐ and inter‐population variation in the travel time taken between smolt detection and arrival in marine conditions. This lag will introduce variability in how the thermal differential indicators represent the sea entry conditions experienced by smolts.

The few significant relationships identified in this study between marine returns and thermal indicators did not hold for detrended time series, suggesting an indirect association between proximal factors that are also impacted by changing thermal environment and smolt migration phenology, such as size and condition of smolts (e.g., Gregory et al., 2019) or the timing and quality of food (Satterthwaite et al., 2014; Tyldesley et al., 2024). This, and the fact that no relationship persisted over all populations, highlights the difficulty in using thermally based metrics as the basis of marine survival indicators for salmon. In general, there appears to be too much regional variation and inconsistency to generalise the use of these indicators across multiple populations.

We conclude that for these salmon populations, thermal differential is not a generally applicable driver or predictor of adult marine return rates and, therefore, would not form a useful basis for an operational indicator of marine survival. Indeed, this is also true for the other thermal indicators based on river and sea entry temperatures examined here.

Climate change poses a significant threat to Atlantic salmon (Gillson et al., 2022). Warming of rivers and seas (IPCC, 2021; Loerke et al., 2023), and the trend towards earlier smolt migration (Teichert et al., 2020), is forecast to continue under current emissions pathways. However, because smolting is primed by photoperiod, the annual cycle of which is invariant, there must be a limit. At some point, salmon populations will be unable to adapt their migration phenology further and may become subject to temperatures beyond their tolerance range, potentially compounded by the increasing frequency of freshwater and marine heatwaves (Bernthal et al., 2023). Thereafter, measures of smolt thermal environment may in the future become proximal indictors of mortality rates.

## AUTHOR CONTRIBUTIONS

All authors contributed to writing and review. Emma Tyldesley: methodology, formal analysis and investigation; Neil Banas and Colin Bull: conceptualisation, methodology, supervision and funding acquisition; Graeme Diack: data curation; Richard Kennedy: data curation and technical advice.

## Supporting information


**DATA S1.** Supporting information.

## Data Availability

The smolt emigration timing data are available from the Knowledge Network for Biocomplexity Repository at https://knb.ecoinformatics.org/view/urn%3Auuid%3Ae3939e37‐bc7c‐488b‐b64d‐43c0494a3593. The return rate data are available from the ICES Working Group on North Atlantic Salmon 2024 report at https://doi.org/10.17895/ices.pub.25730247. The Scottish Shelf Waters Reanalysis Service data are available under the Open Government Licence for public sector information (https://sites.google.com/view/ssw-rs/home).
