# Supplementary material for: River–sea thermal differential experienced by salmon post‐smolts is not a proximal driver of marine survival
Source: J Fish Biol. 2025 Jul 8;107(4):1411–9. doi: 10.1111/jfb.70134 (PMC12536060; doi:10.1111/jfb.70134)
Supplement: Supplementary file 1 — DATA S1. Supporting information. [file JFB-107-1411-s001.docx]

# River-sea thermal differential experienced by salmon post-smolts is not a proximal driver of marine survival: Supporting Information

Table 1 gives details of the river temperature data used for each salmon population. The time series are shown in Figure 1 along with an indication of the overlap with smolt migration time series.

Figure 2 shows time series of adult one sea-winter return rates, smolt migration initiation and the thermal indicators for each population.

Figure 1: River temperature time series.


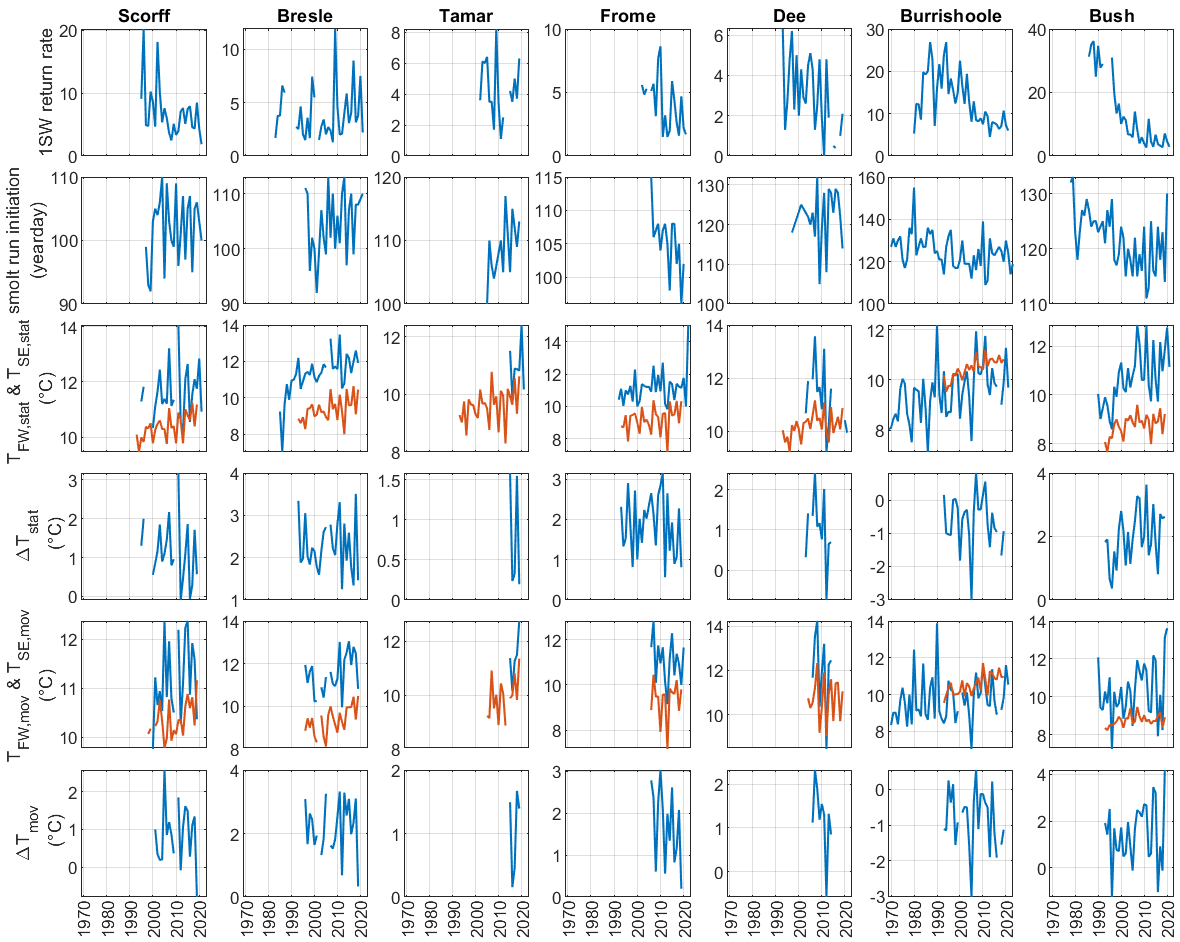


Figure 2: Time series by salmon population of one sea winter (1SW) marine return rate, smolt run initiation and smolt thermal environment indicators: stationary (rows 3 and 4) and non-stationary (rows 5 and 6) freshwater and sea entry (red) temperatures and difference.
